# Lignin and Quercetin Synthesis Underlies Berry Russeting in ‘Sunshine Muscat’ Grape

**DOI:** 10.3390/biom10050690

**Published:** 2020-04-29

**Authors:** Yan Huang, Dong Liang, Hui Xia, Li-Jin Lin, Jin Wang, Xiu-Lan Lv

**Affiliations:** Institute of Pomology and Olericulture, Sichuan Agricultural University, Huimin road 211, Wenjiang district, Chengdu 611130, China; huangyan1994f@163.com (Y.H.); liangeast@sicau.edu.cn (D.L.); susanxia_2001@163.com (H.X.); llj800924@163.com (L.-J.L.)

**Keywords:** phenylpropane synthesis, lignin, quercetin, russet

## Abstract

In order to further explore the mechanism of ‘sunshine muscat’ grape russet formation, transcriptomic and metabolomic analyses were performed on ‘sunshine muscat’ grape peels with and without russet. A total of 1491 differentially expressed genes (DEGs) were discovered based on these analyses. The phenylpropane synthesis pathway was the key metabolic pathway identified, and 28 DEGs related to phenylpropane synthesis pathway were screened, of which 16 were related to lignin synthesis. In addition, 60 differential metabolites were screened. There were 29 phenolic substances among the differential metabolites, which were all up-regulated and 10 were quercetin-related glycosides. Our results indicate that phenols likely play a dominant role in the formation of ‘sunshine muscat’ grape russet, and the synthesis of lignin and quercetin may be the key factors underlying russet formation.

## 1. Introduction

Russet refers to the yellow-brown spots appearing on the surfaces of some fruits during their development. It is a physiological abnormality that occurs as fruits mature and is essentially a layer of secondary protective tissue that serves the function of resistance to adverse environmental conditions and protection of the fruit itself [1]. The problem of russet is prominent in the cultivation of fruit trees, in which it seriously affects the smoothness of fruit surfaces and impairs the quality and the commodity value of fruits. Therefore, this significantly decreases their commercial value [2,3]. In recent years, there have been a number of studies that have focused on apple and pear russet. However, little research has been focused on the grape russet. Researchers have found that cinnamyl-CoA reductase (*CCR*), cinnamyl alcohol dehydrogenase (*CAD*), and peroxidase (*POD*) genes involved in lignin biosynthesis affect russet formation in sand pears and apples [4]. Thus, lignin accumulates to promote the formation of russet by enhancing the expression of genes related to lignin synthesis [5,6]. The formation of apple russet is caused by the rupture of the stratum corneum and the formation of a cork layer. The *MdSHN3* transcription factor is a positive regulator of the formation of cuticles in apple fruits and inhibits the formation of the apple russet [7]. The formation of ‘Dangshansu pear’ russet may be related to phenylpropane metabolism, ethylene metabolism, and secondary metabolism, while the formation of the peel russet may be regulated by lignin synthesis, polyamines, and H_2_O_2_ signals [8]. Studies have shown that the formation of polyphenolic compounds is the main cause of russet formation in ‘sunshine muscat’ grape berries during ripening [9,10]. The synthetic pathway of phenolic compounds is quite complex. First, phosphoenolpyruvate(PEP) and erythrose phosphate (E4P) form phenylalanine through the shikimic acid pathway. Then, under the action of various enzymes, phenylalanine passes through the phenylpropanoid metabolic pathway and flavonoid pathway to form phenolic compounds [11]. Thus far, studies on ‘sunshine muscat’ grape russet formation are very limited, and, until now, the molecular and metabolic pathways engaged in grape russet remain elusive.

With the development of multiple omics technologies, a single omics technique can no longer meet the demands of scientific research, especially research related to plants that have more secondary metabolites. It is difficult to clarify the mechanisms through which environmental changes and other factors impact fruit quality using a single set of omics technologies. In recent years, an increasing number of researchers have applied multiple omics techniques to address research objectives, and the integrative application of multiple omics technologies is becoming more extensive. Genes and metabolites involved in the same biological process will likely exhibit similar changes. Thus, association analysis of transcriptomes and metabolomes is an effective way to find key metabolic pathways and key genes and reveal the underlying molecular mechanisms. A recent study based on metabolomics and transcriptomics revealed the mechanism of peel coloring during jujube maturation, which found that underlying flavonoid metabolites have changed and that changes in structural genes or their regulators (i.e., transcription factors) involved in flavonoid biosynthetic pathways may be the mechanism delaying the accumulation of red anthocyanins in the peels of jujube fruits [12]. This integrated metabolomics and transcriptomics approach has revealed significantly regulated metabolites and biological pathways in citrus fruits, providing new insights into the mechanism of fruit quality deterioration and the induction of resistance against *Penicillium digitatum* in Citrus fruit [13]. Such an analysis has been conducted on the genes and metabolites encoding stilbene synthase (a key enzyme involved in resveratrol synthesis) in grape peel after UV-C irradiation using full transcriptomics sequencing and metabolomics techniques. The anabolism network of astragalus in fruit after UV-A irradiation has been conducted for the first time [14]. 

In order to explore the mechanism of grape russet formation, ‘sunshine muscat’ grapes were examined in this study. Russet and non-russet grapes peel samples were collected at five different stages and mixed uniformly. Transcriptomic sequencing, metabolomic detection, and association of transcriptomic and metabolomics analyses were conducted to screen for the key metabolic pathways and identify differentially expressed genes (DEGs) and metabolites related to russet formation. Knowledge about the physiological and molecular mechanisms underlying ‘sunshine muscat’ grape russet formation will establish a theoretical foundation for the screening of ‘sunshine muscat’ grape russet control measures and high-quality fruit formation. 

## 2. Materials and Methods

### 2.1. Plant Materials

**Figure 1:**
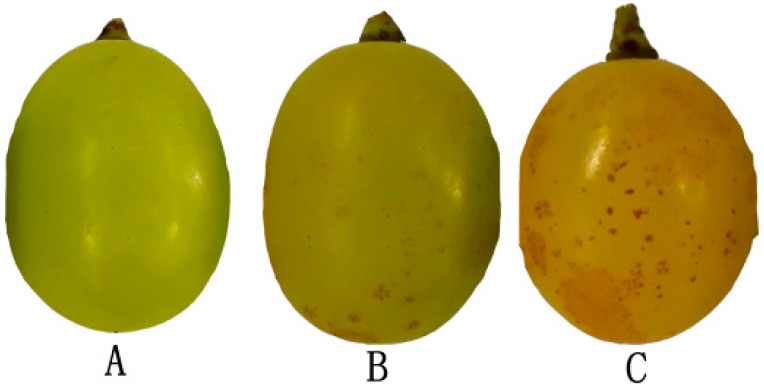
‘Sunshine muscat’ grapes with different degrees of the russet phenotype: (**A**) non-russet grape, (**B**) slightly russet grape, and (**C**) extremely russet grape.

‘Sunshine muscat’ grapes were harvested from a vineyard located at the modern agriculture research and development base of Sichuan Agricultural University (30°33′46″ N, 103°39′36″ E) in July 2019 (the critical period before russet formation). The row spacing of the vineyard was 1.5 m × 3.0 m, and the four-year-old vines were characterized by the uniformity of their vigor and soil and water management in the field. Sampling was performed 70, 80, 90, 100, and 110 days after flowering, and 60–80 berries were collected each time. To study the russet formation mechanism, russet and non-russet grape peels were separated from collected grapes (Figure 1), frozen in liquid nitrogen, and stored at −80 °C in an ultra-low temperature refrigerator until subsequent use. The russet and non-russet grapes peels, which were collected across five stages, were separately and uniformly mixed, and the mixed russet peels (CKY) and mixed non-russet peels (CKN) were used for transcriptomic sequencing and metabolomic assays.

### 2.2. Total RNA Extraction and Sequencing

Grape peels with and without russet were separately mixed at equal quantities from each stage, and RNA was extracted. Each sample consisted of three replicates for RNA-seq sequencing. Grape peels were ground with liquid nitrogen for RNA extraction. The RNAprep Pure Polysaccharide polyphenol plant total RNA extraction kit (Qiagen, Hilden, Germany) was used for RNA extraction, and the constructed sequencing library (prepared with the NEBNext^®^ Ultra™ RNA Library Prep Kit for Illumina^®^ was used to construct the library) was sequenced on the Illumina Hi SeqTM4000 platform (Illumina, San Diego, CA, USA).

### 2.3. Data Quality Control

All raw sequencing reads were first processed with in-house Perl scripts (Novogene Technologies Co., Ltd., Beijing, China). In order to ensure the quality and reliability of data analysis, the original data were filtered through the removal of reads containing adapter sequence, uncalled ‘N’ bases, and too many low-quality bases (i.e., reads for which *Q*_phred_ ≤ 20 bases accounted for more than 50% of the total read length). At the same time, Q20 (the percentage of bases with phred > 20, phred = −10log_10_(e)), Q30 (the percentage of bases with phred > 30, phred = −10log_10_(e)), and GC (the percentage of G and C among bases called in clean reads) content calculations were performed on the clean reads. All subsequent analyses were high-quality analyses based on clean data.

### 2.4. Differentially Expressed Gene Analysis and Enrichment Analysis

RSEM software [15] was used to quantitatively analyze the gene expression levels of each sample. Unigene expression was expressed as fragments per kilobase of exon per million fragments mapped (FPKM) values. The method of Benjamini and Hochberg was used to correct the *p*-values of differentially expressed genes (DEGs) for multiple corrections. The thresholds of differentially expressed unigenes were an absolute fold change >1 and *p* < 0.05 [16], which were used as standards to identify genes that were significantly differentially expressed (where fold change was the FPKM ratio of each unigene between the russet and non-russet grape peel libraries). Cluster Profiler (3.4.4) software was applied to conduct Gene Ontology (GO) enrichment analysis of DEGs and statistical enrichment of DEGs in various Kyoto Encyclopedia of Genes and Genomes (KEGG) pathways.

### 2.5. qRT-PCR Analysis

Sixteen genes involved in lignin biosynthesis were selected to validate the results of RNA-seq by qRT-PCR. The primers used in qRT-PCR are shown in Table 1. The TB Green Premix Ex Taq II (Tli RNaseH Plus, Takara, Beijing, China) was used to perform qRT-PCR. The thermal cycling program for qPCR was 95 °C for 30 s, which was followed by 40 cycles of 95 °C for 5 s and 60 °C for 30 s. *VvGAPDH* was used as the reference gene for normalization. The relative expression levels of the genes were analyzed by the comparative threshold cycle(CT)method (2^−ΔΔCT^ method) [17].

### 2.6. Measurement and Analysis of Metabolomes

Liquid chromatography-mass spectrometry (LC-MS) technology [18,19] based on the highly sensitive SCIEX QTRAP^®^ 6500+ mass spectrometry platform (SCIEX, Framingham, MA, USA) was used to conduct a quasi-targeted metabolomics assay. We collected 100-mg tissue samples, which were frozen in liquid nitrogen in an eppendorf(EP) tube, to which 500 μL of 80% methanol aqueous solution containing 0.1% formic acid was added. Samples were then whirled, shocked, immersed in an ice bath for 5 min, and centrifuged at 4 °C and 15,000 rpm for 10 min. A volume of supernatant was added to a one-half volume of mass spectrometry water diluted to 53% methanol content. Supernatant samples were placed in a centrifuge tube and centrifuged at 4 °C and 15,000× *g* for 20 min. Then, the supernatant was collected for LC-MS analysis. An equal volume of the sample was taken from each experimental sample and mixed for use as a quality control (QC) sample. Based on the Novogene database, the multi-response monitoring model (MRM) was used to assess experimental samples, and the data were analyzed by principal component analysis (PCA), partial least squares-discriminant analysis (PLS-DA), and other multivariate statistical analyses, with the aim of elucidating differences in metabolites between the russet and non-russet grapes.

## 3. Results

### 3.1. Transcriptome Sequencing Yield Statistics

The constructed grape peel transcriptome libraries were sequenced using the high-throughput Illumina platform, obtaining 87,831,088 and 84,645,916 raw reads for the russet CKY and non-russet CKN libraries, respectively. After removing low-quality sequences, 85,425,282 and 82,194,414 clean reads were obtained for the russet CKY and non-russet CKN libraries, respectively. The clean bases totaled 6.45 G and 6.17 G after filtering the CKY and CKN libraries, respectively. Q20 and Q30 sequences comprised more than 95% (sequencing error rate <1%) and 90% of all clean reads, respectively, and the GC content exceeded 45% (Table 2), which indicates that the sequencing data volume and quality were both satisfactory for subsequent sequence assembly and analysis.

### 3.2. Analysis of Differentially Expressed Genes and Gene Enrichment in CKY versus CKN Libraries

**Figure 2:**
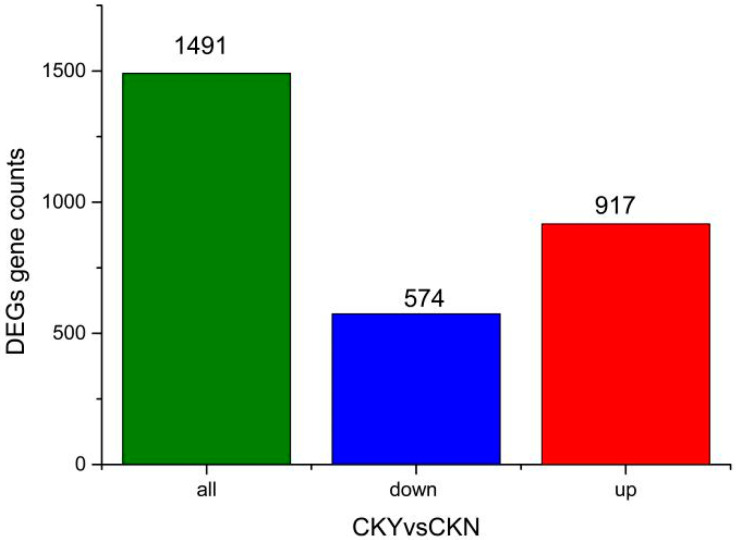
Significantly differentially expressed genes in the russet CKY versus non-russet CKN comparison.

After screening DEGs according to adjusted *p*-values (*P*_adjusted_ < 0.05), a total of 1491 significantly DEGs were identified between the CKY and CKN transcriptomes, of which 574 and 917 genes were down-regulated and up-regulated, respectively, in CKY (Figure 2).

**Figure 3:**
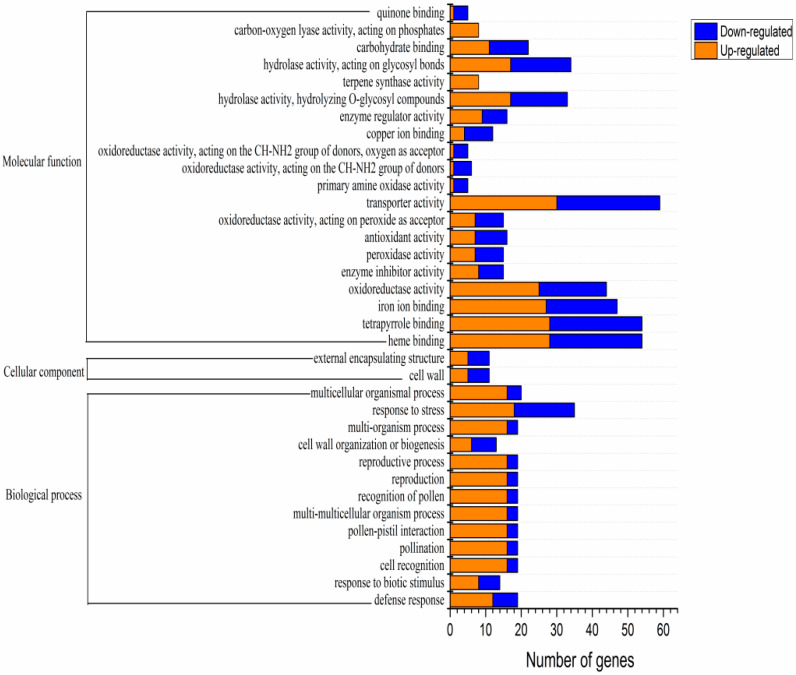
Gene Ontology (GO) annotation classification summary of the russet CKY versus non-russet CKN differentially expressed genes.

Based on the Gene Ontology (GO) database, GO functional clustering was performed on the DEGs obtained by sequencing (*P*_adjusted_ < 0.05). GO classifications are divided into three categories: cell components (CC), biological processes (BP), and molecular function (MF). The identified DEGs provided insights into the molecular mechanisms related to russet formation and were subjected to GO analysis to identify their enrichment in various roles. A total of 35 enriched GO terms were found, including 2 cell components, 13 biological processes, and 35 molecular functions. The CC classification mainly included the overall components of membranes and cell wall morphology. The BP classification mainly included biosynthetic processes and biological stress responses, and the MF classification mainly included oxidoreductase activity, hydrolase activity, antioxidant activity, peroxidase activity, and carbohydrate binding (Figure 3).

Based on the KEGG database, the candidate metabolic pathways related to grape russet formation were identified to further study the biological functions of these genes. As seen in Table 3, the metabolic pathways related to grape russet formation were mainly phenylpropane synthesis, plant hormone signal transduction, and glutathione metabolism. The number of up-regulated DEGs was greater than the number of down-regulated DEGs generally (*P*_adjusted_ < 0.05). The number of genes up-regulated in secondary metabolic pathways, such as phenylpropane synthesis, plant hormone signal transduction, and glutathione metabolism, was significantly greater than that of down-regulated genes, which indicates that the formation of ‘sunshine muscat’ grape russet might be related to these up-regulated genes.

### 3.3. Differentially Expressed Genes Involved in Phenylpropane Synthesis

Differentially expressed unigenes were assessed at thresholds of a corrected absolute log_2_(Fold Change) > 1 and *p* < 0.05 in order to screen genes that were significantly differentially expressed. Analyzing and screening DEGs in the phenylpropane synthesis pathways between CKY and CKN libraries yielded 28 related DEGs of which 17 and 11 were up-regulated and down-regulated, respectively (Table 4). Based on the number of up-regulated and down-regulated genes and the involved pathways, it appears that grape peel russet is connected to the up-regulation of caffeic acid 3-*O*-methyltransferase (100854172), cinnamyl alcohol dehydrogenase (100262421), hydroxycinnamoyl transfer enzyme (100262421), caffeoyl coenzyme AO methyltransferase (100233087), peroxidase (100854817, 100249955, 100242338, and 100262575), 4-coumarin-CoA ligase (100254698), and cinnamyl-CoA ligase (100251623) genes, which are involved in lignin synthesis. Thus, synthesis of lignin in peels might underlie the formation of the grape russet.

### 3.4. Reliability Validation of RNA-seq by qRT-PCR

**Figure 4:**
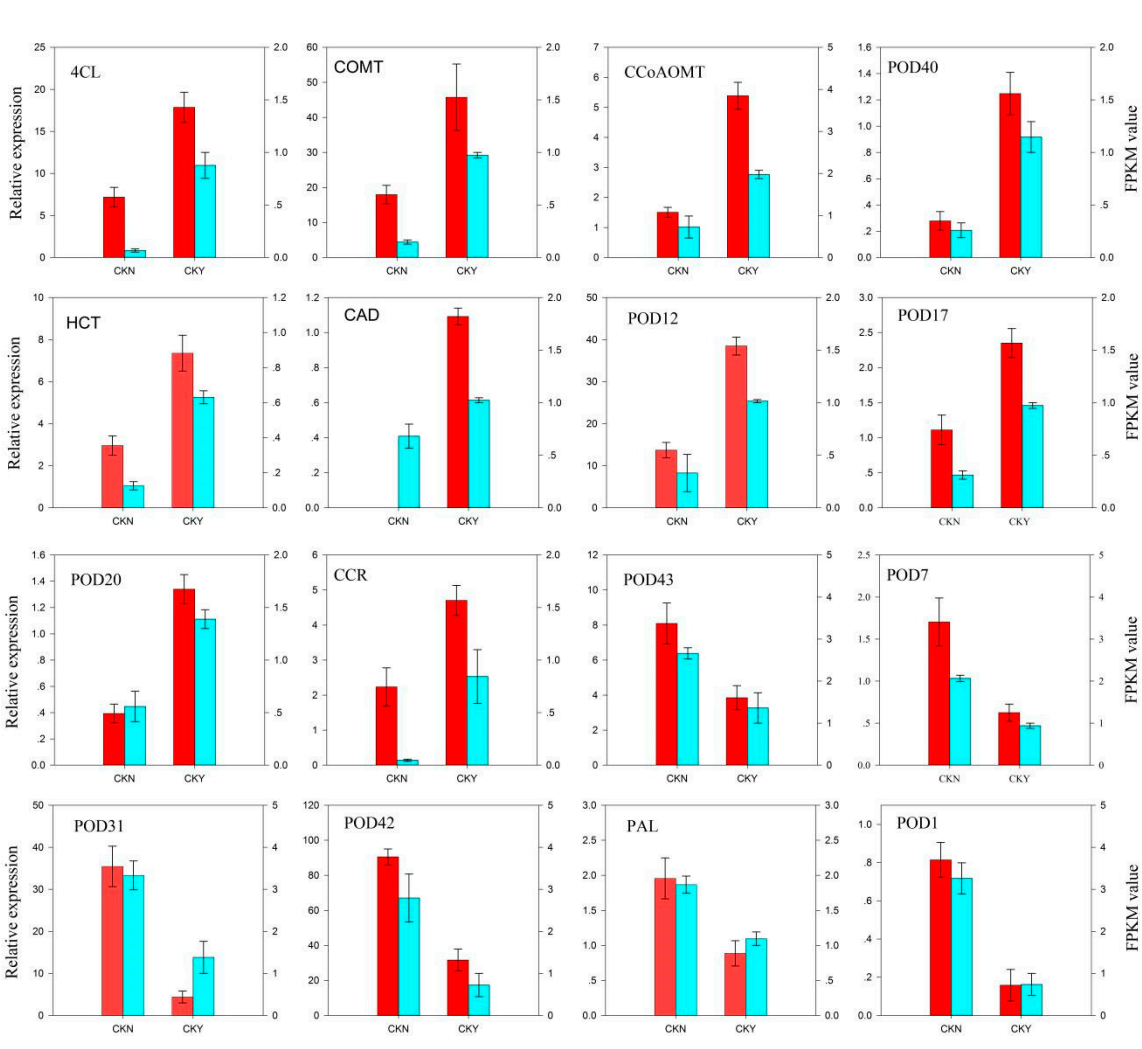
qRT-PCR validation of RNA-seq data. The left *y*-axis shows the relative gene expression levels as assessed by qRT-PCR and the right *y*-axis indicates the corresponding RNA-seq expression data. Each value in the histogram represents the mean ± standard error. CKN and CKY indicate non-russet and russet peel samples, respectively. 4 coumarate CoA ligase, *4CL*, caffeic acid 3-*O*-methyltransferase, *COMT*, cinnamyl alcohol dehydrogenase, *CAD*, shikimate *O*-hydroxycinnamoyl transferase, *HCT*, caffeoyl-CoA *O*-methyltransferase, *CCoAOMT*, peroxidase, *POD*, cinnamoyl-CoA reductase, *CCR*, and phenylalanine ammonia-lyase, *PAL*.

In order to further confirm the reliability and accuracy of Illumina RNA-seq analysis results, 16 key genes involved in lignin biosynthesis were selected, and their expression was evaluated by real-time fluorescent quantitative PCR(qRT-PCR). The expression patterns of these genes obtained from qRT-PCR were highly consistent with those in the RNA-seq data (Figure 4), which indicates that the RNA-seq data were reliable.

### 3.5. Metabolite PCA and PLS-DA

**Figure 5:**
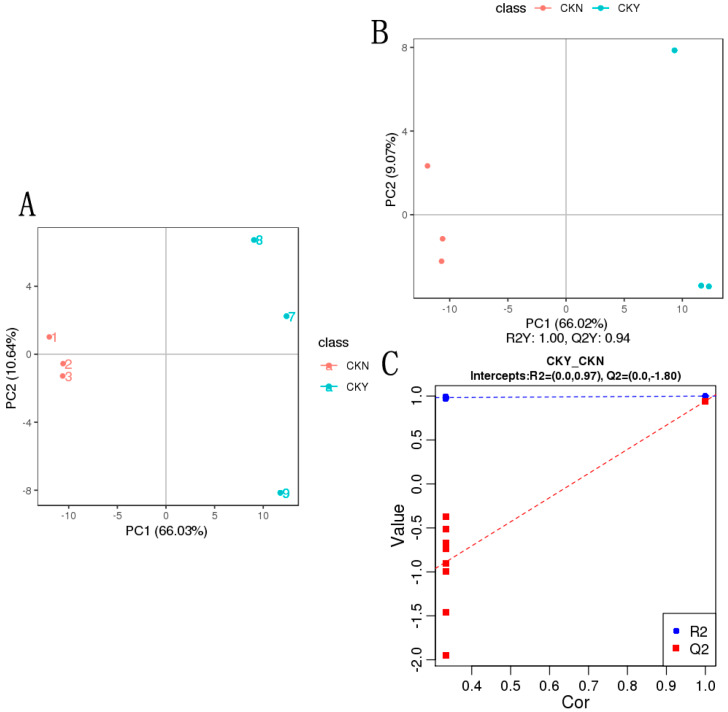
Principal component analysis (PCA) and partial least squares discrimination analysis (PLS-DA) of the CKY versus CKN comparison. PCA (**A**). PLS-DA score map (**B**). Replacement test (**C**).

The multidimensional statistical analysis method principal component analysis (PCA) was conducted to classify the samples. Because no external factors were considered, the obtained PCA model reflected the overall differences in the metabolome data, which clarified the metabolites present in the skins of grapes with and without russet. The distance between the CKY and CKN samples was very great, which indicated a substantial difference between the metabolites in the CKY and CKN treatments (Figure 5A).

Partial least squares discrimination analysis (PLS-DA) utilized partial least squares regression [20] to establish a relationship model between metabolite expression and sample type, which aimed to predict sample types based on the metabolites observed. The PLS-DA models of each comparison group were established, and the model evaluation parameters (*R^2^*, *Q*^2^) were obtained through 7-fold cross-validation. *R*^2^ and *Q*^2^ values closer to 1 indicated a model was more stable and reliable. The results of the PLS-DA score map (Figure 5B) and displacement test for CKY and CKN samples (Figure 5C) were similar to the PCA analysis results. The model coefficients of CKY and CKN samples were *Q*^2^Y = 0.94 and *R*^2^Y = 1.00, which indicated that the model had high predictive ability and goodness-of-fit. In the displacement test, the model *Q*^2^ point was far lower than the rightmost original *Q*^2^ point from left to right. The rightmost *R*^2^ and *Q*^2^ values were greater than 0.9, which indicates that the model had better predictive ability and was effective and usable.

### 3.6. Differential Metabolite Analysis

The variable importance in the projection (VIP) value of the first principal component of the PLS-DA model represents the contribution rate of metabolite differences in different groups, which was assessed [21]. Additionally, the fold change (FC) expresses the ratio of the mean of the repeated quantitative values of all organisms in the comparison group for each metabolite. FC and VIP were combined with *p*-values of *t*-tests between metabolite expression levels to assess metabolites at thresholds of VIP > 1.0, FC > 1.2 (or FC < 0.833), and *p* < 0.05 [22,23,24]. In total, 443 metabolites were screened, which revealed 60 differential metabolites through this analysis, including 43 differentially up-regulated metabolites and 17 differentially down-regulated metabolites (Table 5). Among the 60 different metabolites screened, there were 29 phenolic substances up-regulated as well as 8 nucleic acids and their derivatives, 3 amino acids and their derivatives, and 20 other substances (Table 6).

### 3.7. Analysis of Relative Contents of Key Differential Metabolites

**Figure 6:**
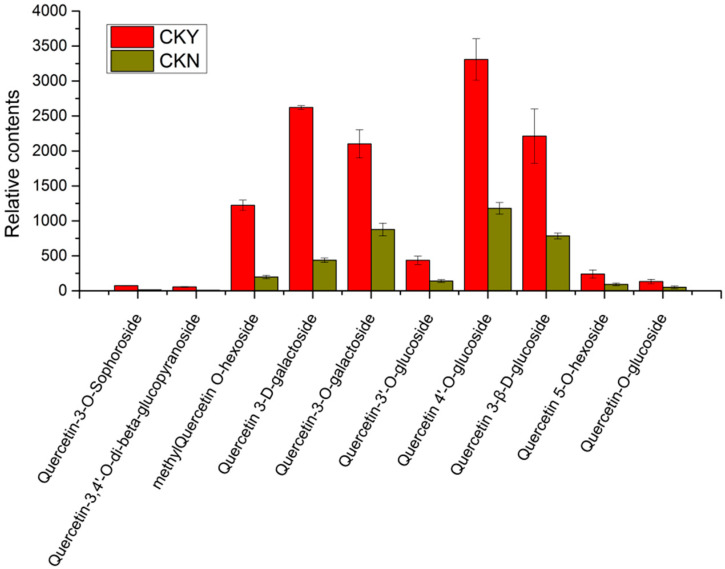
Relative content of key differential metabolites in russet CKY versus non-russet CKN.

A total of 10 quercetin-related glycosides were found among 29 different phenolic metabolites (Table 6), including quercetin glucopyranoside, quercetin-3-*O*-sophoroside, quercetin-*O*-glucoside, quercetin-5-*O*-hexoside, quercetin3-β-d-glucoside, quercetin 4′glucoside, quercetin-3′-*O*-glucoside, quercetin-3-*O*-β-d-galactopyranoside, methylquercetin *O*-hexoside, and quercetin 3-d-galactoside of which methyl quercetin *O*-hexosides, quercetin 3-d-galactosides, quercetin-3-*O*-galactosides, quercetin 4′glucoside, and quercetin 3-β-d-glucoside had higher relative contents of glucosides (Figure 6). Among the 10 quercetin glycosides, the quercetin glycoside content of the CKY sample was significantly higher than that of the CKN sample. Thus, we deduced that the quercetin-related compounds from the phenylpropane metabolic pathway were likely to be the key metabolites involved in the formation of ‘sunshine muscat’ grape russet since quercetin itself is unstable and mostly existed in the form of quercetin glycosides.

### 3.8. Analysis of Different Metabolites and Metabolic Pathways

**Figure 7:**
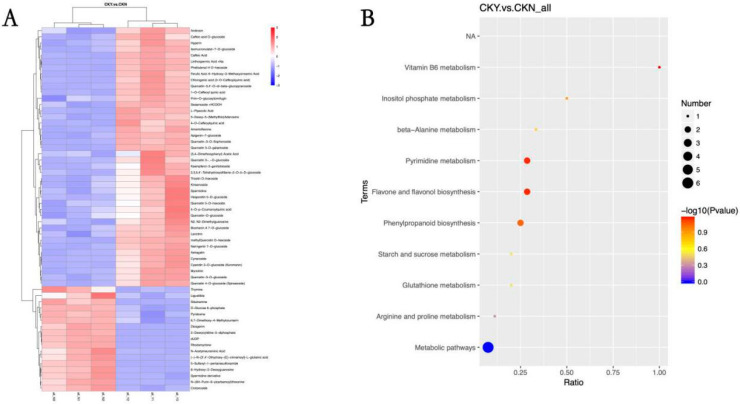
Heat maps of various metabolites clustering (**A**) and metabolic pathway KEGG enrichment bubble map (**B**) of the russet CKY versus non-russet CKN comparison. Sample clustering is shown in the branching diagram at the top of (**A**), while the metabolite clustering is shown in the branching diagram at the left side of (**A**). Shorter branches indicate higher similarity. Red represents up-regulation, while blue represents down-regulation (**A**). The abscissa in (**B**) is *x*/*y* (where *x* is the number of differential metabolites in the corresponding metabolic pathway and *y* is the total number of metabolites identified in the pathway). The colors of the points represent the *p*-values of a hyper geometric test. The smaller the value, the greater the reliability and statistical significance. The size of the point represents the number of differential metabolites in the corresponding pathway. The larger the point, the more differential metabolites occur in the pathway (**B**).

Hierarchical cluster analysis was performed on the differential metabolites between the CKY and CKN samples. After clustering analysis of the identified metabolites, clearly up-regulated or down-regulated metabolites were visible, and the two groups could be distinguished according to metabolite expression (Figure 7A). Phenylpropane biosynthesis, including flavonoid and flavonol biosynthesis, and differential metabolite accumulation of products of pyrimidine metabolism were related to the ‘sunshine muscat’ grape russet phenotype (Figure 7B).

### 3.9. Association Analysis between Transcriptomic and Metabolomic Data

**Figure 8:**
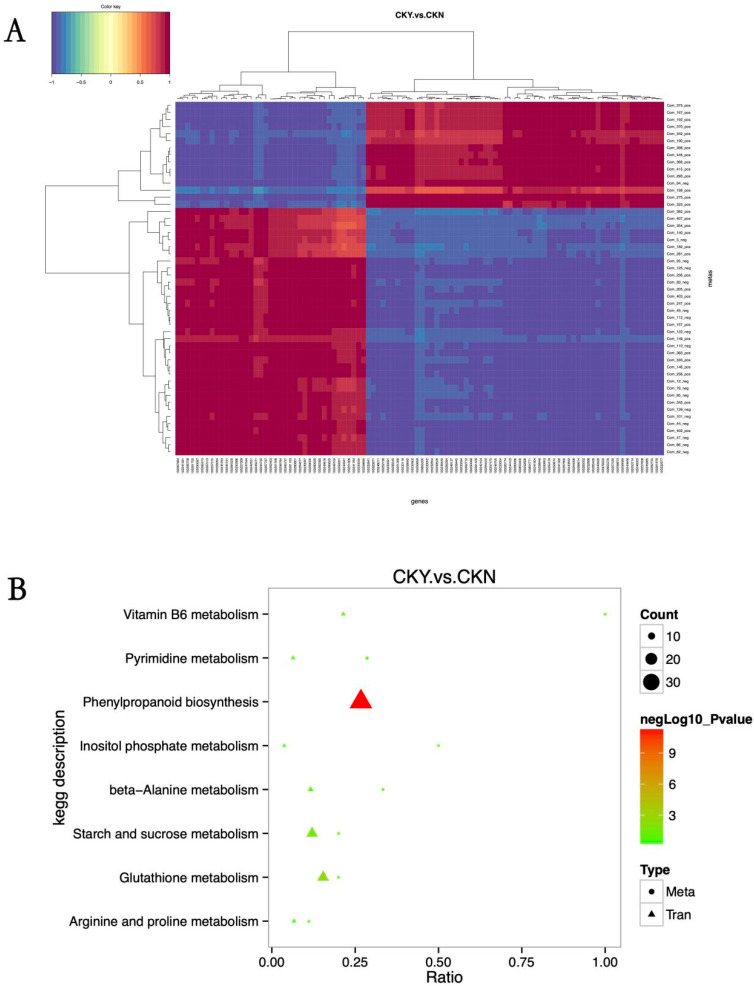
Association analysis between differentially expressed metabolites and genes according to KEGG pathways. Correlation analysis (**A**). Correlation coefficients less than 0 describe negative correlations, shown in blue, while those greater than 0 describe a positive correlation, shown in red. The branching diagram at the top of (**A**) represents clusters of differentially expressed genes, while the branching diagram at the left of (**A**) represents clusters of differentially expressed metabolites. The shorter the clustering branch, the higher the similarity. KEGG pathway analysis (**B**). The abscissa is the ratio of the differentially expressed metabolites or genes enriched in the pathway to the number of metabolites or genes annotated in the pathway (Ratio), and the ordinate is the KEGG pathway enriched according to the metabolome–transcriptome enrichment analysis. Count is the number of metabolites or genes enriched in the pathway.

Association analysis was performed based on the Pearson correlation coefficient between genes that were significantly different in the transcriptomic analysis, and metabolites that were significantly different in the metabolomic analysis were assessed for the degree of correlation between the differential genes and differential metabolites. There was a significant correlation between the DEGs and differential metabolites in ‘sunshine muscat’ grape peels with and without russet (Figure 8A). All the DEGs and metabolites obtained from the russet and non-russet peels of ‘sunshine muscat’ grapes were mapped to the KEGG pathway database to obtain their common pathways and identify the major biochemical pathways and signal transduction pathways in which differentially expressed metabolites and genes were involved. The transcriptomic and metabolomic differences were mainly enriched in phenylpropane metabolic pathways, which is followed by starch and sugar metabolism pathways, and then the glutathione glycine metabolism pathway (Figure 8B).

**Figure 9:**
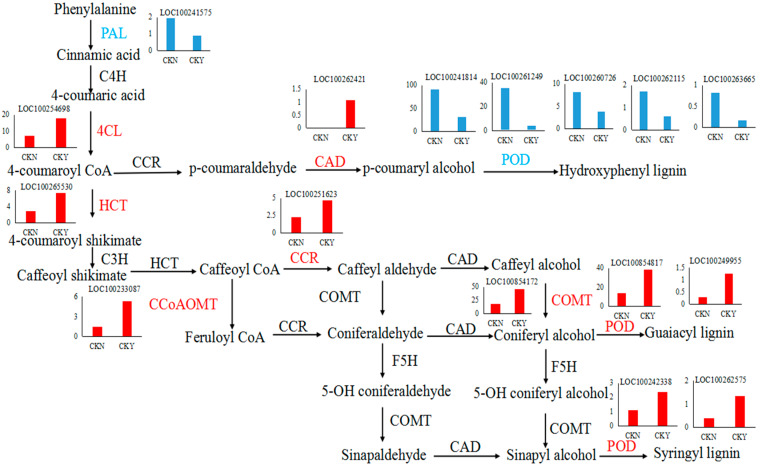
Lignin biosynthesis pathway. Each histogram displays the expression levels of each particular gene (FPKM value). The charts in blue and red represent the down-regulated and up-regulated structural genes, respectively. Phenylalanine ammonia-lyase, *PAL*, cinnamic acid 4-hydroxylase, *C4H*, 4 coumarate CoA ligase, *4CL*, cinnamoyl-CoA reductase, *CCR*, cinnamyl alcohol dehydrogenase, *CAD*, peroxidase, *POD*, shikimate *O*-hydroxycinnamoyl transferase, *HCT*, coumarate-3-hydroxylase, *C3H*, caffeoyl-CoA *O*-methyltransferase, *CCoAOMT*, caffeic acid 3-*O*-methyltransferase, *COMT*, ferulic acid-5-hydroxylase, *F5H*, non-russet, CKN, russet, CKY.

**Figure 10:**
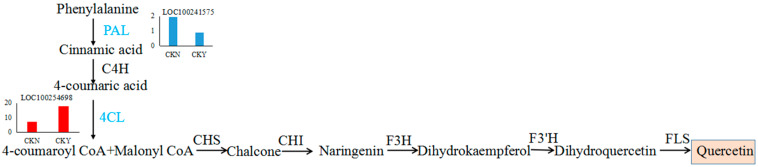
Quercetin biosynthesis pathway. Each histogram displays the expression levels of each gene (FPKM value). The histograms in blue and red represent the down-regulated and up-regulated structural genes, respectively. Chalcone synthase, *CHS*. Chalcone isomerase, *CHI*. Flavanone 3-hydroxylase, *F3H*. Flavanone 3′-hydroxylase, *F3′H*. Flavonol synthase, *FLS*.

Lignin and quercetin are both products of the phenylpropane metabolic pathway. The expression levels of *4CL*, *CAD*, *HCT*, *CCR*, *CCo AOMT*, and *COMT* were significantly higher in russet CKY samples compared with non-russet CKN samples. *POD* expression was observed to be both up-regulated and down-regulated. In addition, *PAL* expression was significantly lower in CKY samples when compared with CKN samples. These genes are all involved in lignin biosynthesis (Figure 9). Moreover, *PAL* and *4CL* are not only involved in lignin biosynthesis but also in the formation of quercetin (Figure 10). Therefore, these genes might be responsible for the ‘sunshine muscat’ grape russet phenotype. Caffeic acid, chlorogenic acid (3-*O*-caffeoylquinic acid), 4-*O*-p-coumaroylquinic acid, and ferulic acid, which are involved in lignin biosynthesis, were up-regulated in russet ‘sunshine muscat’ grapes (Table 6). Naringenin 7-*O*-glucoside and quercetin-related glycosides, which are involved in quercetin biosynthesis, were both up-regulated in russet ‘sunshine muscat’ grapes (Table 6). Our results suggested that lignin and quercetin are the most important metabolites involved in the formation of ‘sunshine muscat’ grapes russet. The genes that encode *4CL*, *CAD*, *HCT*, *CCR*, *CCo AOMT*, *COMT*, *POD*, and *PAL* were most related to russet formation, which suggests that the expression of these genes might induce the accumulation of metabolites related to ‘sunshine muscat’ grape russet. Thus, the results of the transcriptomic and metabolomic analysis were consistent with each other, which indicates that the synthesis of the quercetin and lignin likely underlies the formation of the ‘sunshine muscat’ grape russet.

## 4. Discussion

Thus far, researchers have studied pear and apple russet in detail. Research on ‘Dangshansu pear’ has shown that phenylpropane metabolic pathways are related to the formation of russet, and lignin biosynthesis can regulate russet formation [8,25]. Enzymes involved in lignin biosynthesis can be divided into three categories [26]: (1) enzymes in the phenylalanine metabolic pathway, e.g., *PAL* and *4CL*, which show expression that is closely related to the total lignin, (2) enzymes related to the regulation of lignin monomer synthesis in lignin-specific synthesis pathways, e.g., *COMT* and *CCo AOMT*, (3) enzymes downstream of the lignin synthesis branch pathway and involved in the regulation of enzymes involved in the synthesis and polymerization of lignin monomers, including *CCR*, *CAD*, and *POD*, which are responsible for the ultimate reduction of various hydroxycinnamyl-coenzyme A esters into lignin monomers. The monomers are polymerized into lignin, which play a key role in lignin synthesis and metabolism (Figure 9).

Studies on pears and apples have highlighted that lignin synthesis genes, such as *COMT* and *C4H*, have increased expression, underlying the lignin accumulation that leads to russet formation [5,27]. Studies on ‘Dangshansu Pear’ mutant varieties have shown that increased expression of *CCoAOMT* leads to an increase in the lignin content of the outer peel, which results in russet formation [28]. Similar results were observed in this study. We found that the expression of genes related to lignin synthesis was significantly different between CKY and CKN. Furthermore, we evaluated their expression by qRT-PCR. The expression patterns of these genes obtained from qRT-PCR were highly consistent with those in the RNA-seq data (Figure 4). Thus, these genes may be considered the key genes conferring the formation of the ‘sunshine muscat’ grape russet. Hence, peel lignin synthesis appears to underlie the formation of ‘sunshine muscat’ grape russet (Figure 9). However, the differences in lignin content and the expression of genes related to lignin synthesis during the rusting process in ‘sunshine muscat’ grape require further exploration.

Quercetin (3,5,7,3′,4′-pentahydroxyflavones) is an important aglycon in flavonoids, which are almost insoluble in water, and it is mostly present in the flowers, leaves, and fruit, among other tissues [29,30,31]. Quercetin is an important secondary metabolite whose stress resistance determines its distribution in grape fruits. It is mainly concentrated in the outer epidermal cells of fruits with lower content in the pulp and seeds [32]. Quercetin biosynthesis begins with coumaryl Co A and malonyl Co A, which both form chalcone under the catalysis of chalcone synthase (*CHS*), and chalcone synthesizes naringenin under the catalysis of chalcone isomerase (*CHI*). In turn, naringenin forms dihydro kaempferol under the action of flavonol-3-hydroxylase and then synthesizes dihydroquercetin under the action of flavonoid 3′hydroxylase. Lastly, dihydroquercetin synthesizes quercetin under the action of flavonol synthase [33] (Figure 10). Studies have shown that FaGT6 (DQ289587) can catalyze quercetin to form 3-*O*-glucosidethe in strawberries in addition to a small amount of 7-*O*-, 4′-*O*-, and 3′-*O*- glucosides and other glucosides [34]. Cp3GT (ACS15351) specifically catalyzes glucosylation at the 3-*O* position of quercetin in *Citrus* *paradise* [35]. In grape (*Vitis vinifera*), VvGT5 (AB499074) has quercetin 3-*O*-glucuronyltransferase activity, while VvGT6 (AB499075) uses quercetin as a substrate and has UDP-glucose and UDP-galactose glycotransferase activities [36].

Few reports have explored the relationship between quercetin and quercetin glycosides and the formation of russet. Previous studies on apples have found that the contents of quercetin, quercetin-3-rutin, quercetin-galactoside, quercetin-glucoside, quercetin-xylosin, quercetin-arabinoside, quercetin-rhamnoside, and quercetin-arabin-glucoside in the non-russet bud variety ‘Feng Shuai’ were significantly lower than those in the russet variety ‘Jinguan’, which indicates that the increase in quercetin and quercetin glycosides promotes the formation of ‘Jinguan’ apple russet [37]. The results of this study show that 4-coumarin-CoA ligase (100254698) and quercetin 3-*O*-methyltransferase (100260786) are up-regulated and phenylalanine ammonia-lyase (100241575) is down-regulated in russet ‘sunshine muscat’ grapes (Table 4). The differentially expressed metabolites in the peels with and without russet are mainly phenolic substances, while these phenolic substances are mainly quercetin glycosides. Moreover, the content of quercetin glycosides in russet peel is significantly higher than that in non-russet peel (Figure 6). Therefore, it can be hypothesized that, as quercetin content increases, glycosylation occurs under the action of UDP-glycosyltransferase to form different quercetin glycosylation products during the formation of ‘sunshine muscat’ grape russet. However, changes in quercetin and quercetin glycosides during the rusting process of ‘sunshine muscat’ grape require further exploration.

## 5. Conclusions

We found that the phenylpropane synthesis pathway is the key metabolic pathway associated with russet formation and that the regulation of genes related to lignin and quercetin synthesis differed, which promotes the formation of russet. In addition, phenols are the key metabolites underlying the formation of ‘sunshine muscat’ grape russet. Moreover, lignin and quercetin play major roles in phenol production. Thus, the synthesis of lignin and quercetin is responsible for the formation of berry russeting in ‘sunshine muscat’ grape.

## Figures and Tables

**Table 1:** Gene primers for qRT-PCR.

Gene	Sequence (5′→3′)	Accession Number of Reference Genes Deposited in NCBI
*4CL*	F:GTGTTGGCGATTGCGAAGA R:AGCTTGGCTCTGACAGTGT	XM_002272746.4
*COMT*	F:CCTGGTGTGGAGAATGTTGG R:TTGTCTGGAAGTGCCTGATAAC	XM_003634113.2
*CAD*	F:CGACGGCAAGTTGATTCTCTT R:AGCACTTCCTCTGTCTCCTTC	XM_002285358.4
*HCT*	F:CGCCAGCAAGATCCACAAC R:CGCACCAGAGCCGTTAGAT	XM_002268952.3
*CCo AOMT*	F:AATCATCGGCTACGACAACAC R:GCTCCAACACGAAGTCTCTG	NM_001281118.1
*POD40*	F:CGACATTCACCTCAAGGCTAAC R:AGGCGTCACAAGGTCAAGTT	XM_002273323.3
*POD17*	F:GGGTGTGATGCTTCTTTGTTAC R:TGACTTCTCCAATGCTTCCTTC	XM_002271047.3
*POD12*	F:GCTTGCTTCGCCTCCACTT R:TTCTTGTTCACCAGGACCACTT	XM_003634474.3
*POD20*	F:GGATGCGATGCCTCTATTCTTC R:TGCCTCTTCTACCAAGTGCTT	XM_002279172.4
*CCR*	F:CCACACTGCTTCTCCTGTCA R:GCCGCTGCTATTATCACATTCT	XM_002273418.3
*POD42*	F:GCCAAGAGCCAAGACTACTTC R:GTGCCAGTGAGAGGATTGTTC	XM_002274733.3
*POD31*	F:GCAGGATACCATCACCAACAAG R:AGGAGACAAGAACGGAAGCAT	XM_002280511.3
*POD43*	F:GCTGATATGCCTGATGTGAGTG R:GCTGTGGTTCCAATGGTGTG	XM_002270914.4
*POD7*	F:AAGCAGAGGTTGAGAAGAGGT R:AATGAGGACGGTGGCATCT	XM_002265631.3
*POD1*	F:GTGAACACGGCAGTGAACAA R:TGTCGTCCAGCAGGATTGAT	XM_010655057.2
*PAL*	F:CCAAGGATACTCAGGCATCAGA R:GAGGCAAGCAAGGACTAATGTT	XM_002285241.3
*VvGAPDH*	F:TTCTCGTTGAGGGCTATTCCA R:CCACAGACTTCATCGGTGACA	GU585870

4 coumarate CoA ligase, *4CL*, caffeic acid 3-*O*-methyltransferase, *COMT*, cinnamyl alcohol dehydrogenase, *CAD*, shikimate *O*-hydroxycinnamoyl transferase, *HCT*, caffeoyl-CoA *O*-methyltransferase, *CCoAOMT*, peroxidase, *POD*. cinnamoyl-CoA reductase, *CCR*, phenylalanine ammonia-lyase, *PAL*.

**Table 2:** Summary of sequenced reads.

Sample	Total Raw Reads	Total Clean Reads	Clean Bases	GC Percentage	Q20 Percentage	Q30 Percentage
CKY CKN	87,831,088 84,654,916	85,425,282 82,194,414	6.41 G 6.17 G	46.26% 46.87%	97.58% 97.65%	93.03% 93.27%

CKN and CKY indicate non-russet and russet peel samples, respectively.

**Table 3:** Metabolic pathways significantly enriched for differentially expressed genes (DEGs) in the russet CKY versus non-russet CKN comparison.

KEGG ID	Metabolic Pathway	Number of DEGs	*p*-Value	Up-Regulated Gene Counts	Down-Regulated Gene Counts
vvi00940	Phenylpropanoid biosynthesis	31(11.40%)	7.22 × 10^−12^	20	11
vvi00904	Diterpenoid biosynthesis	7(2.57%)	3.38 × 10^−06^	4	3
vvi00950	Isoquinoline alkaloid biosynthesis	8(2.94%)	9.05 × 10^−06^	4	4
vvi00350	Tyrosine metabolism	11(4.04%)	1.82 × 10^−05^	5	6
vvi00196	Photosynthesis - antenna proteins	6(2.21%)	3.92 × 10^−04^	1	5
vvi00960	Tropane, piperidine, and pyridine alkaloid biosynthesis	7(2.57%)	7.32 × 10^−04^	2	5
vvi00480	Glutathione metabolism	14(5.15%)	2.65 × 10^−03^	8	6
vvi04075	Plant hormone signal transduction	23(8.46%)	4.24 × 10^−03^	19	4

**Table 4:** Differentially expressed genes involved in phenylpropane synthesis.

Gene ID	log_2_(FPKM_CKY_/FPKM_CKN_)	*p*-Value	Gene Description	Up- or Down-Regulated
100267863	2.31	1.60 × 10^−16^	cytochrome P450 84A1	up
100854817	1.49	5.41 × 10^−15^	peroxidase 12	up
100854172	1.35	2.57 × 10^−12^	caffeic acid 3-*O*-methyltransferase	up
100253961	1.64	1.53 × 10^−08^	berberine bridge enzyme-like 26	up
100250740	1.01	1.46 × 10^−07^	berberine bridge enzyme-like 15	up
100262421	6.95	1.64 × 10^−07^	probable cinnamyl alcohol dehydrogenase 1	up
100265530	1.31	4.31 × 10^−07^	shikimate *O*-hydroxycinnamoyltransferase	up
100233087	1.84	6.10 × 10^−06^	caffeoyl-CoA *O*-methyltransferase	up
100261642	3.98	1.03 × 10^−04^	probable mannitol dehydrogenase	up
100249955	2.15	1.18 × 10^−04^	peroxidase 40	up
100265092	2.77	2.36 × 10^−04^	anthocyanidin 3-*O*-glucosyltransferase 5	up
100260786	1.89	3.24 × 10^−04^	quercetin 3-*O*-methyltransferase 1-like	up
100242338	1.08	8.54 × 10^−04^	peroxidase 17	up
100254698	1.31	9.87 × 10^−04^	4-coumarate--CoA ligase	up
100262575	1.75	1.05 × 10^−03^	peroxidase 20	up
100250160	2.21	1.24 × 10^−03^	beta-glucosidase 12	up
100251623	1.07	1.64 × 10^−03^	cinnamoyl-CoA reductase 1	up
100855376	−1.74	1.15 × 10^−32^	probable mannitol dehydrogenase	down
100241814	−1.51	1.47 × 10^−17^	peroxidase 42	down
100261249	−3.01	1.83 × 10^−14^	peroxidase 31	down
100854583	−1.19	5.62 × 10^−13^	probable mannitol dehydrogenase	down
100247559	−2.10	1.56 × 10^−07^	probable mannitol dehydrogenase	down
100252642	−1.70	7.76 × 10^−07^	probable mannitol dehydrogenase	down
100260726	−1.07	3.99 × 10^−06^	peroxidase 43	down
100854646	−1.98	1.15 × 10^−05^	probable mannitol dehydrogenase	down
100262115	−1.45	6.79 × 10^−05^	peroxidase 7	down
100241575	−1.14	2.52 × 10^−04^	phenylalanine ammonia-lyase-like	down
100263665	−2.35	1.17 × 10^−03^	cationic peroxidase 1	down

FPKM indicates fragments per kilobase of exon per million fragments mapped values.

**Table 5:** Differential metabolite level analysis results.

Compared Samples	Num. of Total Ident.	Num. of Total Sig.	Num. of Total Sig. Up	Num. of Total Sig. Down
CKY versus CKN	443	60	43	17

CKN and CKY indicate non-russet and russet peel samples, respectively.

**Table 6:** Significantly different metabolites in the russet CKY versus non-russet CKN comparison.

Number	Metabolites	VIP	FC	*p*-Value	Up- or Down-Regulated
	**Phenols**				
1	Caffeic Acid	1.76	3.63	0.00003	up
2	1-*O*-Caffeoyl quinic acid	2.07	4.56	0.00008	up
3	Isomucronulatol-7-*O*-glucoside	1.23	2.45	0.00112	up
4	4-*O*-Caffeoylquinic acid	1.57	3.16	0.00116	up
5	Chlorogenic acid (3-*O*-Caffeoylquinic acid)	2.02	4.43	0.00162	up
6	Naringenin 7-*O*-glucoside	2.87	8.02	0.0021	up
7	Astragalin	1.43	2.87	0.00316	up
8	Myricitrin	1.27	2.55	0.004	up
9	Cynaroside	1.56	3.16	0.00522	up
10	Amentoflavone	3.35	11.00	0.00631	up
11	Cyanidin 3-*O*-glucoside (Kuromanin)	1.51	3.05	0.00815	up
12	ε-Viniferin 2	2.30	5.23	0.01003	up
13	Kaempferol-3-gentiobioside	3.12	8.91	0.01074	up
14	Hesperetin 5-*O*-glucoside	1.50	3.07	0.01526	up
15	Biochanin A 7-*O*-glucoside	2.63	6.13	0.02243	up
16	Laricitrin	1.06	2.14	0.02356	up
17	4-*O*-p-Coumaroylquinic acid	1.31	2.61	0.01148	up
18	Ferulic acid	2.44	6.03	0.00234	up
19	Caffeic acid *O*-glucoside	1.49	2.97	0.00134	up
20	Quercetin-3,4′-*O*-di-beta-glucopyranoside	2.59	6.62	0.00008	up
21	Quercetin-3-*O*-Sophoroside	2.39	5.74	0.00001	up
22	Quercetin-*O*-glucoside	1.36	2.60	0.04101	up
23	Quercetin 5-*O*-hexoside	1.29	2.60	0.0182	up
24	Quercetin 3-β-d-glucoside	1.40	2.82	0.00861	up
25	Quercetin 4′-*O*-glucoside (Spiraeoside)	1.29	2.59	0.00171	up
26	Quercetin-3′-*O*-glucoside	1.54	3.10	0.00147	up
27	Quercetin-3-*O*-galactoside	1.19	2.40	0.00103	up
28	Methylquercetin *O*-hexoside	2.50	6.20	0.00041	up
29	Quercetin 3-d-galactoside	2.45	5.98	0.00068	up
	**Nucleic acids and their derivatives**				
30	dUDP(2′-deoxyuridine 5′-diphosphate)	5.65	0.02	0	down
31	2′-Deoxycytidine-5′-diphosphate	5.55	0.02	0.00004	down
32	8-Hydroxy-2-Deoxyguanosine	2.41	0.17	0.00018	down
33	*N*-(9H-Purin-6-ylcarbamoyl)threonine	2.30	0.18	0.00057	down
34	5′-Deoxy-5′-(Methylthio)Adenosine	1.23	2.46	0.0016	up
35	Crotonoside	2.43	0.17	0.00481	down
36	*N*2,*N*2-Dimethylguanosine	1.23	2.44	0.01038	up
37	Thymine	1.06	0.46	0.01272	down
	**Amino acids and their derivatives**				
38	l-Pipecolic Acid	1.23	2.45	0.00008	up
39	*N*-Acetylneuraminic Acid	1.29	0.39	0.00897	down
40	(-)-*N*-[3′,4′-Dihydroxy-(E)-cinnamoyl]-l-glutamic acid	1.33	0.37	0.01037	down
	**Others**				
41	Diosgenin	3.82	0.06	0.00003	down
42	Rhodomyrtone	3.40	0.08	0.00006	down
43	5-Sulfanyl-1-pentanesulfonamide	3.22	0.09	0.0001	down
44	6,7-Dimethoxy-4-Methylcoumarin	1.16	0.44	0.02876	down
45	Spermidine derivative	2.31	0.19	0.00193	down
46	d-Glucose 6-phosphate	1.19	0.42	0.00937	down
47	2,3,5,4′-Tetrahydroxystilbene-2-*O*-β-d-glucoside	1.37	2.77	0.01094	up
48	Sibutramine	5.84	0.02	0.01287	down
49	Spermidine	1.25	2.50	0.01395	up
50	Ligustilide	1.08	0.45	0.01732	down
51	Sesamoside +HCOOH	1.69	3.33	0.02052	up
52	Pyridoxine	1.09	0.46	0.02286	down
53	(3,4-Dimethoxyphenyl) acetic acid	1.30	2.64	0.03423	up
54	Androsin	1.51	2.84	0.03835	up
55	Kinsenoside	1.53	3.05	0.00821	up
56	Lithospermic acid +Na	3.53	13.20	0.00003	up
57	Prim-*O*-glucosylcimifugin	1.08	2.13	0.04191	up
58	Apigenin-7-glucoside	3.21	8.93	0.02621	up
59	Phellodenol H *O*-hexoside	1.37	2.74	0.00008	up
60	Tricetin *O*-hexoside	1.39	2.78	0.00623	up

VIP indicates the variable importance in the projection value. FC indicates the fold change.
